# Peri-conceptional changes in maternal exposure to sewage sludge chemicals disturbs fetal thyroid gland development in sheep

**DOI:** 10.1016/j.mce.2012.12.022

**Published:** 2013-03-10

**Authors:** Sabine Hombach-Klonisch, Adrian Danescu, Farhana Begum, Maria R. Amezaga, Stewart M. Rhind, Richard M. Sharpe, Neil P. Evans, Michelle Bellingham, Corinne Cotinot, Beatrice Mandon-Pepin, Paul A. Fowler, Thomas Klonisch

**Affiliations:** a Dept. of Human Anatomy & Cell Science, University of Manitoba, Winnipeg, Canada; b Dept. of Obstetrics, Gynecology & Reproductive Sciences, University of Manitoba, Winnipeg, Canada; c Centre for Reproductive Endocrinology and Medicine, Institute of Medical Sciences, University of Aberdeen, Foresterhill, Aberdeen, AB25 2ZD, UK; d The James Hutton Institute, Craigiebuckler, Aberdeen, AB15 8QH, UK; e MRC Human Reproductive Sciences Unit, The Queen’s Medical Research Institute, University of Edinburgh, 47 Little France Crescent, Edinburgh, UK; f Institute of Biodiversity, Animal Health and Comparative Medicine, College of Medical, Veterinary and Life Sciences, University of Glasgow, Glasgow G61 1QH, UK; g INRA, UMR 1198, Biologie du Developpement et de la Reproduction, 78350 Jouy-en-Josas, France; h Dept. of Medical Microbiology & Infectious Diseases, University of Manitoba, Winnipeg, Canada; i Dept. of Surgery, University of Manitoba, Winnipeg, Canada

**Keywords:** ECs, environmental chemicals, EDCs, endocrine-disrupting compounds, NIS, sodium-iodide symporter, fT3, free triiodothyronine, fT4, free thyroxine, TH, thyroid hormone, TSH, thyroid stimulating hormone, TR, thyroid hormone receptor, TTR, transthyretin, HPT, hypothalamic-pituitary-thyroid axis, PCBs, polychlorinated biphenyls, PBDE, polybrominated diphenyl ether, DEHP, di(2-ethylhexyl) phthalate, CV, coefficient of variation, DAB, 3,3′-diaminobenzidine tetrahydrochloride, HRP, horseradish peroxidase, RT, room temperature, HE, hematoxylin-eosin, GnRH, gonadotropin releasing hormone, GD, gestational day, TUNEL, terminal deoxynucleotidyl transferase dUTP nick end labeling, Endocrine disruptors, Thyroid gland, Sheep, Fetal, Sewage sludge, Development

## Abstract

Ewes were exposed to sewage sludge-fertilized pastures in a study designed investigate pre-conceptual and/or gestational exposure to environmental chemicals. The in utero impact on fetal thyroid morphology and function at day 110 (of 145) of pregnancy was then determined.

Pre-conceptual exposure increased the relative thyroid organ weights in male fetuses. The number of thyroid follicles in thyroids of fetuses after pre-conceptual or gestational exposure was reduced. This correlated with an increase in Ki67 positive cells. Pre-conceptual exposure to sewage sludge reduced small blood vessels in fetal thyroids. Thyroid tissues of exposed fetuses contained regions where mature angio-follicular units were reduced exhibiting decreased immunostaining for sodium-iodide symporter (NIS). Fetal plasma levels of fT3 and fT4 in exposed animals, however, were not different from controls suggesting compensatory changes in the thyroid gland to maintain homeostasis in exposed fetuses. The regional aberrations in thyroid morphology may impact on the post-natal life of the exposed offspring.

## Highlights

► We used an ovine prenatal exposure model to a mixture of environmental chemicals. ► Male fetal thyroids of mixed exposure groups have reduced follicle counts. ► Fetal thyroids of animals in mixed exposure groups show increased proliferation. ► Thyroid glands of exposed fetuses showed regions with impaired differentiation. ► Fetal plasma levels of free thyroid hormones were normal.

## 1 Introduction

Multiple organ systems including the adrenal, thyroid, mammary and pituitary glands, gonads and other parts of the reproductive tract can be adversely affected by developmental exposure to environmental chemicals (ECs), including heavy metals and endocrine-disrupting compounds (EDCs) (Woodruff and Walker, 2008). Over 100 naturally occurring and synthetically derived substances, including many EDCs (e.g., halogenated arylhydrocarbons, bisphenols, phthalates and pesticides), are either suspected or known to have thyroid-disrupting properties and their ability to interfere with normal thyroid development, thyroid hormone (TH) levels, and thyroid function has important implications for animal and human health (Boas et al., 2006, 2009; Crofton, 2008; Kohrle, 2008; Zoeller, 2007). The thyroid gland is a major regulator of cellular metabolic programming and an intact hypothalamic-pituitary-thyroid (HPT) axis is essential for normal pre- and post-natal neuronal and reproductive development and functions (Gauger et al., 2007). Prenatal exposure to polychlorinated biphenyls (PCBs) has been associated with poor mental performance and altered motor functions in children and monkeys (Jacobson and Jacobson, 2003; Levin et al., 1988; Tilson et al., 1979; Walkowiak et al., 2001; Wilhelm et al., 2008). Prenatal exposure to PBDEs was shown to be associated with long-lasting hyperactivity, hearing impairment, and impaired learning and memory (Goldey et al., 1995; Kuriyama et al., 2005). The mechanisms through which ECs interact with the HPT axis are complex, occur at various levels, and the effects may be dependent on the developmental state of exposed cells and tissues in this regulatory circuit. ECs have been reported to alter thyroid homeostasis by blocking iodide uptake and enzymatic inhibition of thyroid peroxidase and deiodinase activity in follicular thyrocytes (Dohan et al., 2007; Gauger et al., 2007; Jugan et al., 2010; Mastorakos et al., 2007; Schmutzler et al., 2007). Some ECs have also been reported to cause the selective and competitive displacement of THs from their receptors and the three main TH binding proteins in serum (Brouwer et al., 1998; Hallgren and Darnerud, 2002; Lema et al., 2008; McKinney and Waller, 1994; Schriks et al., 2007). Detailed reviews on the interaction of EDCs with thyroid physiology have been published (Jugan et al., 2010; Schmutzler et al., 2007) (Boas et al., 2012; Kohrle, 2008; Zoeller, 2007).

Work with specific classes of ECs has documented possible thyroid dependent mechanisms through which specific developmental effects might be mediated. For example, PCBs have a structure similar to TH and can bind to TH receptors (Fritsche et al., 2005; McKinney and Waller, 1994). Hydroxylated PCBs were shown to decrease T4 hormone levels by competing with the binding to the TH-binding protein transthyretin (TTR) (Brouwer et al., 1998). Similar in structure to PCBs, the newly emerging polybrominated diphenyl ether (PBDE) flame retardants and para-hydroxylated PBDE metabolites, and isopropylidenediphenol or bisphenol A can also bind to and antagonize TH receptors (Kitamura et al., 2005; Kojima et al., 2009) (Moriyama et al., 2002; Zoeller et al., 2005), as does di(2-ethylhexyl) phthalate (DEHP) (Ishihara et al., 2003).

In addition to direct effects such as those illustrated above, EDCs can also have secondary effects on health as many ECs are able to induce the expression of the hepatic xenobiotic metabolizing sulfotransferases (SULTs) and uridine diphosphate glucuronyltransferases (UDGPTs) (Brouwer et al., 1998; Nishimura et al., 2005), which through the production of reverse-T3 (rT3) from sulfated T4 can influence TH signaling (Mol and Visser, 1985). Such an EC-induced effect could be developmentally important as plasma T3 concentrations in the ovine fetus remain low for most of gestation because T4 is largely metabolized to biologically inactive rT3 and sulphated TH derivatives and placental enzymes inactivate T3 (Polk, 1995). It is only in the latter part of pregnancy when preferential deiodination of T4 to T3 occurs and this results in higher plasma T3 levels in the ovine fetus near term (Forhead et al., 2006; Polk, 1995).

While studies of the effects of individual chemicals can be important mechanistically, environmental exposure is normally to a complex mixture of chemicals. We have been working with an ovine model in which pregnant animals have been exposed to environmental concentrations of a mixture of EDCs through grazing pastures fertilized with human sewage sludge. Sheep provide an ideal model to study the effects of EC exposure on HPT development and function as, like humans, sheep are long-lived and have a relatively long gestation period of 145 days, during which timing and sequence of organogenesis is similar to that seen in humans. Despite the relatively low concentrations of individual chemicals that are found in the environment and tissues collected from animals maintained in this experimental paradigm (Rhind et al., 2005a,b), including concentrations in the tissues of animals used in the current study (Rhind et al., 2009, 2010), we have reported exposure effects on multiple organ systems including the fetal hypothalamus and pituitary (Bellingham et al., 2009, 2010), fetal gonads (Fowler et al., 2008; Paul et al., 2005), adult gonads (Bellingham et al., 2011; Fowler et al., 2012), offspring behavior (Erhard and Rhind, 2004) and adult bone structure (Lind et al., 2009, 2010).

In the present study, we have investigated the effects of pre-conceptual, gestational, and continuous maternal exposure to sewage sludge on fetal thyroid gland development and the levels of circulating thyroid hormones in the exposed ovine fetus.

## 2 Materials and methods

### 2.1 Animals and treatments

**Fig. 1:**
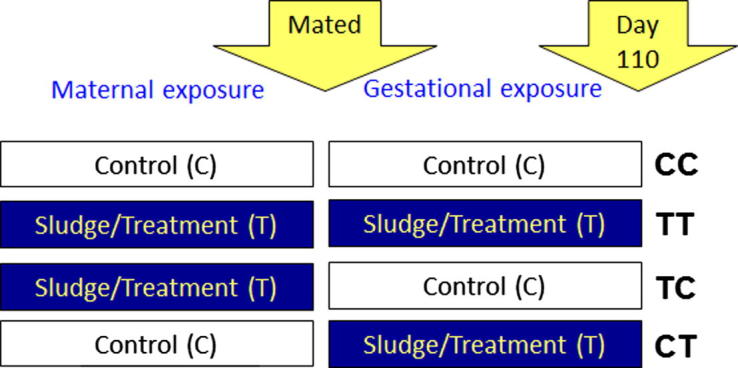
Schematic illustration of the four treatment groups. Groups of sheep were exposed to sewage sludge fertilized pastures at different stages: throughout life before and after mating (TT); only until mating, but not thereafter (TC); only between mating and euthanasia (CT); controls (CC) were always exposed to pasture treated with inorganic fertilizer. Animals were euthanized at GD 110 (of 145 days to term) of pregnancy.

The study was approved by the James Hutton Institute’s Local Ethical Committee and fully licensed by the United Kingdom’s Animals (Scientific Procedures Act 1986) under Project License PPL 60/4028. Animals and exposure regimens to sewage sludge were described earlier (Rhind et al., 2010). Briefly, sheep derived from a single flock of Texel ewes were maintained on pastures treated twice annually with thermally dried digested sewage sludge (2.25 metric tonnes of dry matter per hectare; Treated: T) or with inorganic fertilizer containing equivalent amounts of nitrogen (225 kg per hectare per year; Control: C). Sewage sludge contained numerous EDCs, among them PCBs, phthalates, PAHs, PBDEs, and the analysis was reported previously for a similar batch of sludge (Rhind et al., 2009) and for the soil levels for treated and untreated plots (Rhind et al., 2010). Groups of sheep were exposed to the respective treatments before or after mating or continuously (Fig. 1): exposure of sheep throughout their lives before and after mating (TT) resulting in pre-conceptual and gestational exposure in the fetuses; exposure only until mating, but not thereafter (TC) resulting in exclusively pre-conceptual exposure; exclusively gestational exposure was achieved by exposure between mating and euthanasia (CT). Control animals (CC) were exposed only to pasture treated with inorganic fertilizer, throughout their lives. Pregnant ewes were euthanized at day 110 of gestation, i.e. in the last trimester of pregnancy. Thyroid glands of maternal ewes and fetal lambs were collected within 15 min (min) of euthanasia and their wet weight determined prior to fixation of one lobe in 10% buffered formalin and snap-freezing of the other lobe in liquid nitrogen. The sizes and weights of the fetuses were determined at the time of euthanasia and the thyroid weights expressed relative to the body mass of the fetuses.

### 2.2 Detection of thyroid hormones and thyrotropin (TSH)

Maternal and fetal blood samples were obtained at euthanasia and plasma was separated and stored at −20 °C. Plasma concentrations of free triiodothyronine (fT3) and free tetraiodothyronine (thyroxine, fT4) were measured with the automated ADVIA Centaur® XP competitive immunoassay system (Siemens Healthcare Diagnostics, Camberley, UK), which uses direct chemiluminescent detection. The fT3 inter-assay CV was 3.08% and assay sensitivity 0.3 pmol/L; the fT4 mean intra- and inter-assay CV were 4.00% and 2.23%, respectively, and assay sensitivity was 1.3 pmol/L. Maternal plasma concentrations of TSH were determined using a commercially available ultrasensitive ELISA Kit specifically to detect sheep TSH (U-TSH, Amsbio, Abingdon, UK). Following the manufacturer’s instructions, standards (ranging from 0 to 5 mIU/L) and samples were prepared and added (50 μl) in duplicate to the pre-coated microtiter plate. 100 μl of the horseradish peroxidise (HRP) enzyme conjugate was added to each well including blank control wells which contained PBS alone and incubated for 1 h at 37 °C. After incubation, the plate was washed 5 times using wash buffer. All wells were then incubated with the HRP substrate solution (100 μl) for 10 min at 37 °C before addition of the stop solution (50 μl) to all wells to stop the enzyme reaction. The optical density (OD) was determined at 450 nm using a microplate reader. Blank control readings were subtracted from all mean readings and a standard curve was plotted of TSH concentration (*X* axis) against OD (*Y* axis). Maternal plasma concentrations were calculated using linear regression analysis. Limit of detection was 0.01 mIU/L. Intra assay coefficient of variation was <9%.

### 2.3 Morphometric analysis

Thyroid tissue sections (5 μm) were cut from paraffin embedded fetal tissue. Every 10th section was stained with hematoxylin-eosin (HE) and imaged with a Zeiss Axio Imager A2 microscope using Axiovision 4.8 software (Zeiss, Jena, Germany). For quantitative analysis, the number and the size of follicles, the epithelial height and the area of blood vessels were determined using the Axiovision AutoMeasure quantitative software (Zeiss). Results from male and female fetuses were considered both separately and together. The follicles were assessed in a semi-automated system and manual correction was performed for each slide. Due to the irregularity in size and shape, the blood vessel area was traced manually, and the samples from 10 animals from each treatment group (five males and five females) were analyzed quantitatively. Blood vessel area in the four treatment groups and in the different size categories were compared between groups. For quantitative assessment of follicle numbers and size distribution, a total of 96 animals were assessed: TC group: 26 animals; 13 females, 13 males; CT group: 21 animals (11 females and 10 males); TT group: 25 animals (12 females and 13 males) and CC group: 24 animals (15 females and nine males). Two sections separated by 100 μm were assessed for each animal. Since follicle sizes varied within the same gland and between animals, we classified follicles in four size categories based on surface area: smaller than 150 μm^2^, between 150 and 500 μm^2^, and larger than 500 μm^2^.

### 2.4 Immunohistochemistry

Three 5 μm sections from each animal were used for the immunodetection of Ki67 and NIS (sodium-iodide-symporter). Sections were dewaxed and rehydrated in Tris-based saline containing 0.05% Tween-20 (TBST). Endogenous peroxidase was inactivated with 3% H_2_O_2_ in methanol for 20 min at room temperature (RT). Antigen retrieval was achieved by boiling the sections in citrate buffer (pH 6.0) for 20 min and non-specific binding sites were saturated with TBST containing 5% goat normal serum. For the detection of proliferating cells, sections were incubated at 4 °C overnight with a mouse monoclonal Ki67 antibody (Novocastra, Dossenheim, Germany) diluted 1:100 in blocking buffer. For the detection of NIS protein, a mouse monoclonal antibody (NIS VJ2, generously provided by Dr. S. Costagliola, Université Libre de Bruxelles, Belgium) was used at 1:10 and 1:25 dilutions at 4 °C overnight. This antibody is directed against the extracellular loop of human NIS which shows some sequence conservation with the sheep. A mouse isotype control immunoglobulin (Sigma Aldrich, St. Louis, MO) substituting for the primary antibody served as negative control. For both protocols, a biotinylated goat anti-mouse secondary antibody (Vector Laboratories, Burlingame, CA) was used at 1:200 for 1 h at RT prior to incubations with the Vectastain ABC complexes (Vectastain ABC kit, Vector Laboratories). Specific binding was visualized using the 3,3′-diaminobenzidine tetrahydrochloride (DAB) substrate (Pierce Biotechnology, Rockford, IL). Nuclei were counterstained with hematoxylin and Ki67-positive and NIS-positive cells were imaged using a Zeiss Axio Imager A2 microscope and analysis was performed with the Zeiss Axiovision 4.8 software for male and female thyroids separately. For quantification of Ki67-positive nuclei, five non-overlapping images from each thyroid section were taken at 200× magnification. The number of Ki67-positive and the total number of hematoxylin-stained nuclei was determined semi-quantitatively (Zeiss Axiovision AutoMeasure software with manual correction) and analyzed separately for male and female fetal thyroids.

### 2.5 TUNEL assay

Apoptosis was detected using the TumorTacs in situ apoptosis detection assay (Trevigen, Gaithersburg, MD) according to the manufacturer’s instructions. Briefly, sections were dewaxed, rehydrated, treated with proteinase K solution and quenched with 3% hydrogen peroxide solution. The 3′ ends of cleaved DNA fragments in apoptotic cells are recognized by the terminal deoxynucleotidyl transferase for the incorporation of biotinylated nucleotides. Visualization of chromosomal DNA fragments was facilitated by binding of streptavidin-HRP and subsequent diaminobenzidine (DAB) treatment. Sections treated with nuclease to induce DNA fragmentation served as positive control for apoptosis detection. A nuclear counterstain was performed with methyl green.

### 2.6 Statistical analysis

Normality of data distribution was tested with the Shapiro–Wilk test and non-normally distributed data were log-transformed prior to analysis using one-way ANOVA. For the Ki67 immunodetection and the relative thyroid organ weights, statistical analysis was performed with the ANOVA test. For the morphometric analysis of the follicle number, follicle size and blood vessel area, a two-factor analysis of variance and a chi-square analysis were employed to determine differences for treatment and gender. Analyses of endocrine data were performed using JMP (5.1, Thomson Learning, London, UK).

## 3 Results

### 3.1 Relative thyroid weights are increased in male fetuses of the TC group

No statistically significant effects of EC exposure were seen on either maternal or fetal thyroid gross weights (Table 1). In addition, no statistically significant sex differences were observed within the fetal animals in each treatment group (Table 1). However, when thyroid weights were normalized in relation to body weight, those in the control male fetuses were significantly (*p* = 0.013) lighter than those of control females. Also, normalized thyroid weights of male fetuses were significantly (20%; *p* = 0.038) increased in the TC group compared to those of controls.

### 3.2 Fetal thyroid hormone levels are not changed

Fetal plasma concentrations of free T3 (fT3) and free T4 (fT4) and the fT3/fT4 ratio were not altered significantly by treatment. However, fT3 concentrations were reduced in the CT dams and fT4 concentrations were reduced in CT and TC dams relative to CC animals (Table 1). There were no significant treatment effects on the maternal fT3/fT4 ratios (Table 1). The plasma TSH levels in the dams were not significantly altered between the four groups (Table 1) although the increase of TSH levels in the TT compared to the CT group showed borderline significance (*p* = 0.06): CC vs. CT: *p* = 0.10; CC vs. TC: *p* = 0.23; CC vs. TT: 0.85; CT vs. TC: *p* = 0.66; CT vs. TT: *p* = 0.06; TC vs. TT: *p* = 0.16. No significant correlation of any kind was found between maternal TSH and fetal thyroid hormone levels.

### 3.3 Thyroid morphological changes suggest delayed differentiation in exposed animals

**Fig. 2:**
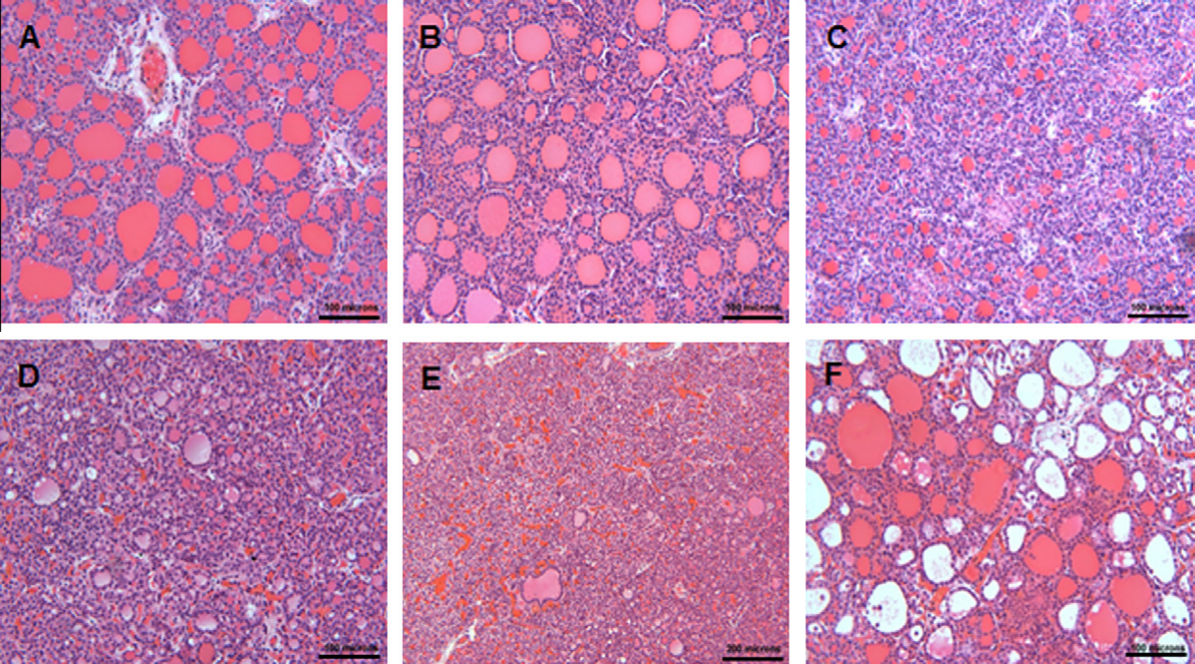
Representative H&E staining of fetal thyroid glandular tissues from different treatment groups: controls (A), CT (B), TC (C), and TT (D–F). The intra-individual variability was most marked in the TT-treatment group as shown here for two animals as examples (D and E from the same animal; F from a different animal within the TT treatment group). Magnification ×200; scale bar = 100 μm.

**Fig. 3:**
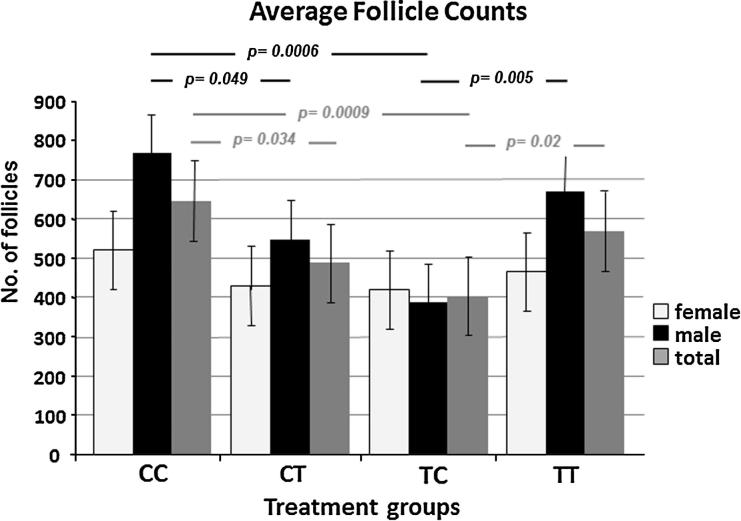
Differential effects of sewage sludge exposure on fetal sheep thyroid follicle numbers. The significant treatment pairs were found in males (CC vs. CT, *p* = 0.049; CC vs. TC, *p* = 0.0006; TC vs. TT, *p* = 0.005). Females followed the same trend and, when both genders were considered together, changes were still significant for the total animals. Significant differences were observed between both cross-over exposure groups vs. the controls and between the TC and TT groups. Statistical analysis was performed using two-factor ANOVA. Means ± SEM are given.

**Fig. 4:**
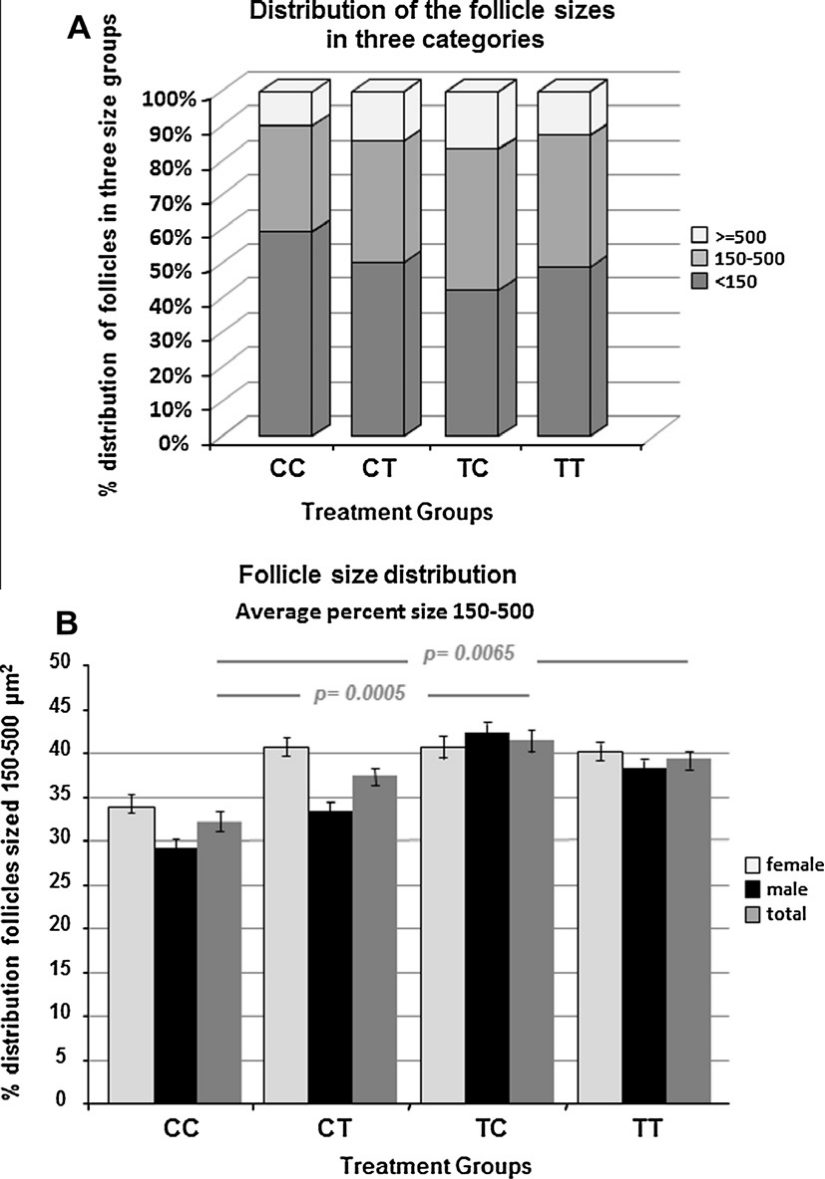
Differential effects of sewage sludge exposure on fetal sheep thyroid gland follicle size categories. The follicles were categorized in three size groups: (1) <150 μm^2^, (2) 150–500 μm^2^, and (3) >500 μm^2^ using the Axiovision AutoMeasure quantitative software (Zeiss). Statistical significance was determined with the Chi-Square analysis. Only the 150–500 μm^2^ category of thyroid follicle area showed significant differences between treatment groups: the percent distribution of 150–500 μm^2^ follicles in the TC and TT treatment groups was different from controls (CC). We observed a trend for controls (CC) vs. CT-treated animals. Means ± SEM are given.

We performed histological image analysis of thyroid glands from control, CT, and TC animals (Fig. 2A–C), and three different images of thyroid morphology observed in the TT group (Fig. 2D–F). There was considerable inter-animal variability which was most pronounced in the TT group. Quantitative analysis of the thyroid follicle numbers revealed significant differences in the mean average thyroid follicle count with gender (*p* = 0.009) and treatment (*p* = 0.007). Treatment-induced differences were statistically significant only in male fetuses (CC vs. CT, *p* = 0.049; CC vs. TC, *p* = 0.0006; TC vs. TT, *p* = 0.005), although a similar pattern of change was seen between the four groups in females (Fig. 3). Specifically, the significant reduction in follicle numbers compared to controls occurred exclusively in the TC and CT treatment groups, and the TC group compared to the TT group (Fig. 3). We determined follicle sizes between groups and observed that only the middle size category (150–500 μm^2^) showed significant differences between treatment groups. The percent distribution of 150–500 μm^2^ follicles in the TC and TT treatment groups was significantly higher (*p* = 0.0005 and 0.0065, respectively) compared to controls (CC), and there was a trend (*p* = 0.0568) for the control (CC) group and the CT-treated animals (Fig. 4). In summary, the cross-over treatment groups presented with a lower follicle count, and both groups with preconceptual exposure showed a higher percentage distribution of medium-sized follicles. The height of the follicular epithelium was unchanged and we did not observe alterations in the follicular resorption vacuoles.

**Fig. 5:**
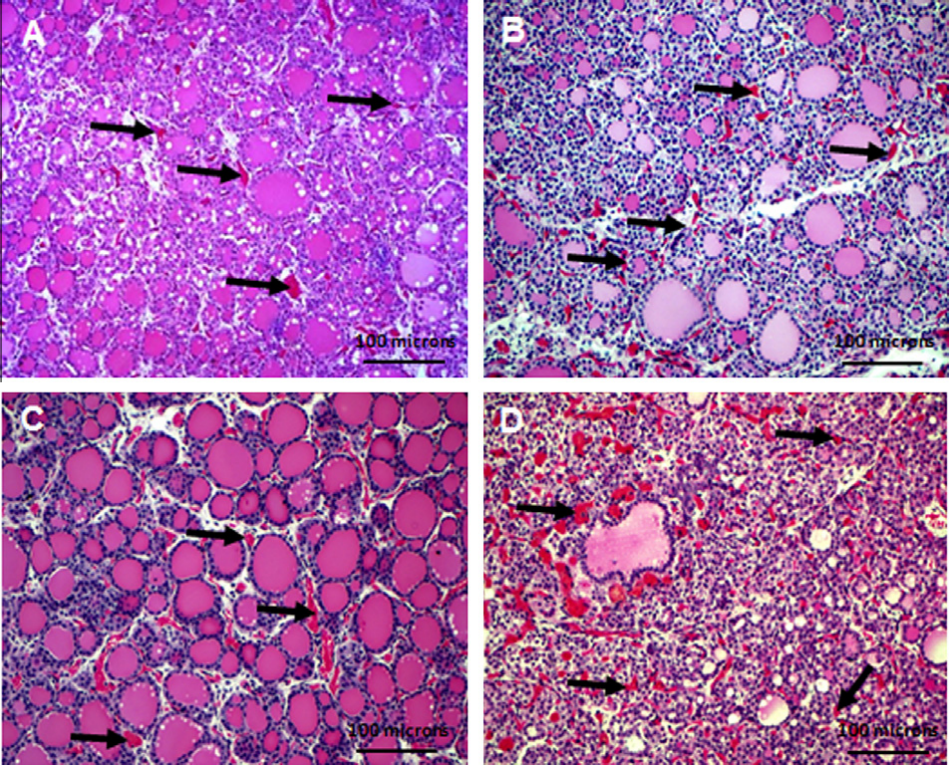
Representative H&E staining of fetal thyroid glandular tissue from different treatment groups: controls (A), CT (B), TC (C), and TT (D). Arrows depict examples of blood vessels which were outlined manually for the quantitative assessment of blood vessel area for each section in controls (CC; A), CT (B), TC (CI), and TT (D). Magnification ×200; scale bar = 100 μm.

**Fig. 6:**
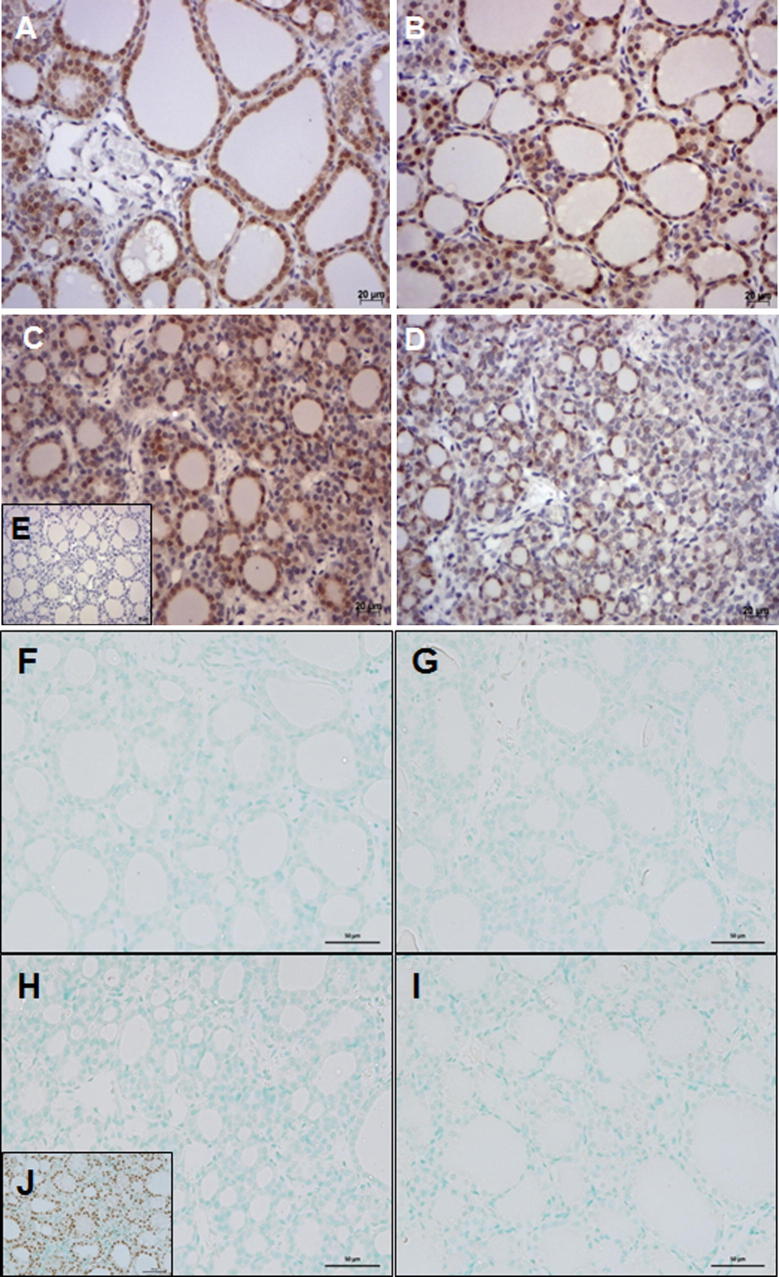
Representative images of the detection of NIS protein (A–E) and in situ apoptosis (F–I) in thyroid sections of animals from the different treatment groups: controls (CC; A), CT (B), TC (C), and TT (D). Immunoreactive NIS protein was present exclusively in thyroid follicular cells and the expression was strongest in morphologically well-differentiated thyroid follicles. The isotype control antibody did not yield any staining (E). Nuclei were counterstained with hematoxylin. Representative images for the in situ TUNEL test are shown for the CC (F), CT (G), TC (H), and TT (I) groups and demonstrate the lack of apoptotic nuclei in all groups. Nuclease-treated sections served as positive control and show brown nuclear staining (insert J). Magnification: ×400; marker bar = 20 μm (A–E); scale Bar = 50 μm (F–J).

We determined the blood vessel area (Fig. 5) in a subset of fetuses (10 animals per treatment; five animals of each sex). There were no significant differences in the mean total blood vessel area with treatment (*p* = 0.57) or sex (*p* = 0.09). However, we observed significant changes in the relative distribution of blood vessel sizes (Table 2). Smaller blood vessels (<100 μm^2^) representing more than 80% of all blood vessels in the thyroid tissues (Table 3) were predominantly affected. In females, the mean area of these small blood vessels was significantly reduced in the TT fetal thyroids compared to all other groups (TT vs. CC, *p* < 0.001; TT vs. CT, *p* < 0.002; TT vs. TC, *p* < 0.004). Male fetuses showed a significant reduction in small blood vessels in the TC group relative to CC (*p* < 0.003), CT (*p* < 0.001), and TT animals (*p* < 0.001). Thus, while female fetuses were most affected with constant exposure in the TT group, male fetuses were most affected with only preconceptual exposure in the TC group. In addition, we observed regions lacking follicular organization of thyrocytes and showing numerous dilated blood vessels (Fig. 5D). These regions of immature thyroid follicle development and impaired angio-follicular units were exclusively observed in the TC and TT treatment groups in both male and female fetuses. The intricate relationship between polarized follicular thyroid epithelial cells and the surrounding capillary network is essential for thyroid function and referred to as “angio-follicular unit” (Gerard et al., 2002, 2000), the smallest functional unit in the thyroid gland. Thyroid cell differentiation was determined by immunodetection of the sodium-iodide transporter protein (NIS). Strong expression of NIS protein, indicating thyroid cell differentiation, was detected in thyroid follicular cells of large and small follicles but not in thyroid cells lacking follicular organization (Fig. 6D; TT group). This reduction in NIS expression coincided with the absence of morphological features of thyroid follicular organization of thyrocytes in both, male and female fetuses.

### 3.4 Fetal thyroids show increased proliferation and no apoptosis in exposed animals

**Fig. 7:**
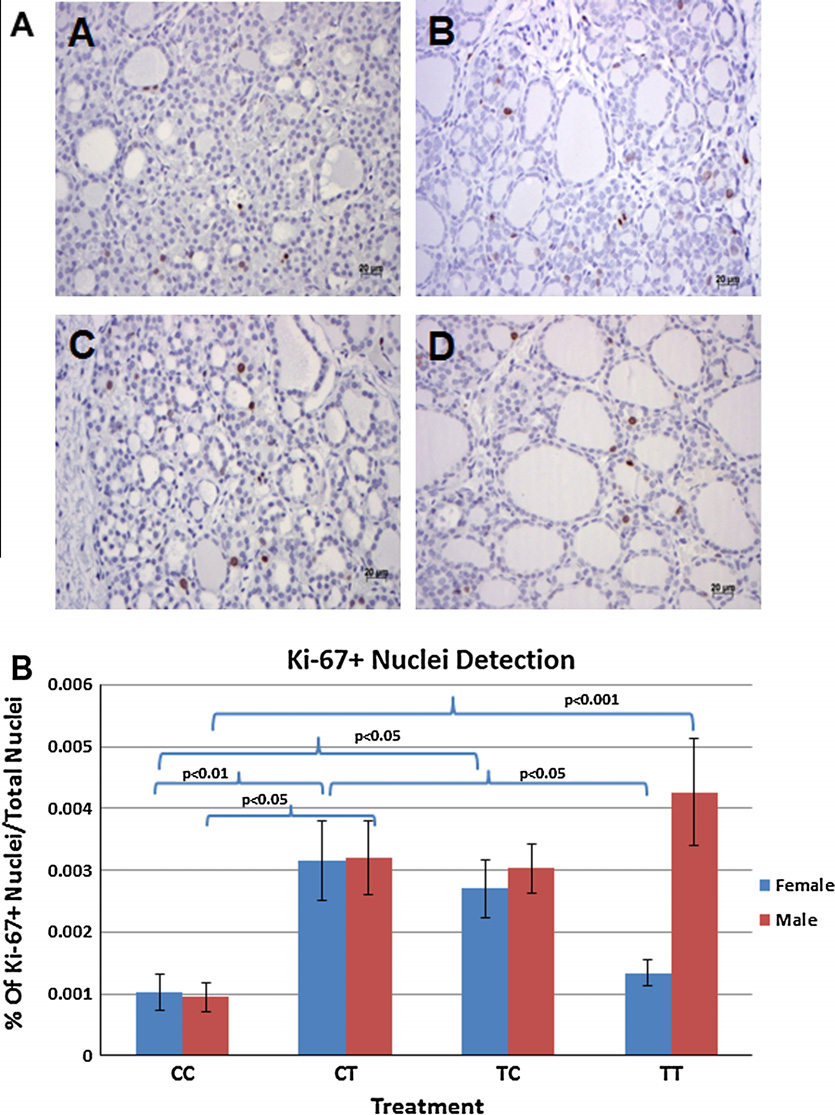
Differential effects of pre-conception and/or in utero sewage sludge exposures on fetal sheep thyroid gland cell proliferation. (A) Representative images from Ki67-positive immunostaining on thyroid sections from fetal sheep of different treatment groups: controls (CC; A), CT (B), TC (C), and TT (D). Magnification: ×400; scale bar = 20 μm. (B) In females, significant differences in proliferation were found between both cross-over treatment groups and the controls (CC vs. CT, *p* < 0.01; CC vs. TC, *p* < 0.05) and between the CT and TT group (*p* < 0.05). In males, significant differences in proliferation were found between the CC and CT group (*p* < 0.05) and the CC and TT group (*p* < 0.001): ANOVA and Tukey HSD test was performed. Means ± SEM are given.

To assess the balance between apoptosis and proliferation in the fetal thyroids following pre-conceptual and/or gestational exposure to sewage sludge fertilized pastures, we determined TUNEL-positive apoptotic nuclei and Ki67-positive nuclei of proliferative cells. We failed to detect apoptotic nuclei in the majority of fetal thyroid tissues of all treatment groups (Fig. 6F–I), with the exception of very few and isolated apoptotic nuclei in one or two animals of each group. Individual Ki67-positive thyroid cells were detected in very low numbers throughout the thyroid sections indicative of cell proliferation (Fig. 7A) and quantitative analysis showed differences between male and female fetuses. In females, we detected a significant increase in Ki67-positive nuclei in both cross-over groups (CT vs. CC: *p* < 0.01; TC vs. CC: *p* < 0.05; CT vs. TT: *p* < 0.05) (Fig. 7B). Male fetuses showed the same trend for both cross-over groups (significant only for CC vs. CT: *p* < 0.05) and higher proliferation in the TT group (TT vs. CC: *p* < 0.001) (Fig. 7B) when compared to controls.

In summary, the key findings from the histological analysis of the fetal thyroid glands were the reduction in average follicle numbers in both cross-over groups in males, the changes in follicle size distribution in the TC and TT groups, the reduced area of small blood vessels in the TC and TT group and the presence of regions with impaired thyroid differentiation in angio-follicular units in the TC and TT groups.

Female fetuses consistently showed a reduction in the percentage of small blood vessel after pre-conceptual exposure (TC, TT) and an exclusive and significant increase in thyroid cell proliferation in both cross-over groups (CT, TC). Male fetuses showed significantly reduced follicle counts in both cross-over groups (CT, TC) and revealed highest thyroid cell proliferative activity in the TT group.

## 4 Discussion

Our study clearly demonstrates that exposure of an out-bred species to a complex mixture of ECs and EDCs at environmentally-relevant concentrations exerted adverse effects on the maternal and fetal thyroid systems. We conclude that peri-conceptual changes in exposure most strongly affected thyroid gland development by: (i) reducing circulating maternal fT3 and fT4 concentrations, (ii) increasing fetal thyroid gland weight in males, and (iii) disrupting aspects of fetal thyroid morphology, notably follicle number and size, size distribution of blood vessels, and rate of proliferation. Despite those changes, fetal fT3 and fT4 levels were largely unaffected suggesting effective homeostatic regulation of the fetal thyroid system.

While maternal thyroid weights were unaltered by treatment, plasma levels of free thyroxine (fT4) in the ewes at gestational day (GD) 110 were lower in TC and CT groups and plasma levels of free triiodothyronine (fT3) concentrations were lower in the CT group than in controls. However, maternal TSH levels did not show significant changes between treatment groups and there was no correlation between maternal TSH and fetal THs. We conclude that ewes of the CT and TC groups presented with a condition resembling very mild hypothyroidism. Interestingly, this reduction in maternal fT4 plasma levels was exclusively observed in the cross-over treatment groups, but not in the TT animals, suggesting that the change in exposure to the environmental chemicals affected TH homeostasis rather than a steady exposure. Maternal THs reach the fetal circulation via the placenta, and their contribution to fetal TH homeostasis is crucial during embryo (Kaplan, 1994; Patel et al., 2011; Zoeller et al., 2007) and brain (Cooper, 2005; Mastorakos et al., 2007; Porterfield and Hendrich, 1993) development. At the beginning of the third trimester of pregnancy, the fetal hypothalamo-pituitary-thyroid (HPT)-axis is functional (Obregon et al., 2007). Importantly, the transplacental transfer of maternal THs is insufficient to maintain fetal thyroid homeostasis as shown for sheep fetuses with fetal thyroidectomy at GD 105/110 which resulted in a dramatic reduction in fetal plasma T3 and T4 concentrations at GD 130 (Forhead et al., 2009). Our study shows that fetal TH levels at GD 110 were normal and the fetal thyroid histology did not provide any indication of TSH-induced thyroid hypertrophy, such as increased epithelial height. However, we cannot exclude the possibility that the fetuses were affected by a potential transient hypothyroid state of the mothers during early fetal life.

Significant changes observed in the mixed exposure groups (CT and TC) included decreased follicle counts in males, increased number of Ki67-positive cells in female fetuses and an increase in relative thyroid weights in males. This suggests that some aspects of fetal thyroid gland structural organization are affected by the change in exposure to real-life ECs through the mothers rather than by gestational exposure itself. We did not detect any signs of increased apoptosis in the fetal thyroids of any group demonstrating that programmed cell death did not occur in thyrocytes of exposed animals. The observed increased proliferation may indicate an enhanced regenerative activity in females of the cross-over groups (Doganay et al., 2005). Furthermore, a continuous exposure to ECs (TT group) may allow for compensatory mechanisms to develop, such as induction/activation of maternal and foetal enzymatic systems by EDCs from the sludge during gestation. Thus, pre-conceptual exposure may have triggered increased capacity to metabolise and degrade some of the EDCs contained in sewage sludge, e.g., through the actions of the cytochrome P450 system, the UDP-glucuronosyltransferase and the glutathione S-transferase systems, prior to conception, whereas animals of the CT group had de novo exposure at conception. Evidence for our assumption is based on the observation that upon oral exposure tissue concentrations of the PCBs 126 and 153 in sheep during pregnancy were elevated in the mother, fetus and lamb, yet the half-life of PCBs was shortened in animals with prior exposure to high PCB doses and hydroxylated PCBs were detected in higher amounts in exposed sheep suggesting an induced hepatic metabolism of PCBs (Berg et al., 2010). However, at slaughter, no significant differences in fetal or maternal hepatic EC burdens were observed in the same animals whose thyroids were used for this study (Rhind et al., 2010).

Morphological and physiological parameters significantly altered exclusively in the TC and TT treatment groups include a reduction in the percentage of small blood vessel in females, highest thyroid cell proliferative activity in the TT group in males, changes in follicle size distribution in the TC and TT groups, the reduced area of small blood vessels in the TC and TT group and the presence of regions with impaired thyroid differentiation in angio-follicular units in the TC and TT groups. TC and TT exposure groups have in common a pre-conceptual exposure of the mothers to sewage sludge ECs. Our results suggest that pre-conceptual exposure affected distinct aspects of the structural development of the fetal thyroid gland. The formation of the thyroid primordium at embryonic day 10 (E10) in the human embryo and the start of the functional differentiation in week 10 of development are both independent of TSH (Klonisch et al., 2009). It is tempting to speculate that alterations in maternal TH metabolism (Brouwer et al., 1998) or maternal EC body burden during early phases of thyroid development contribute to thyroid programming and to the structural changes detected in ovine fetuses at GD110. Thus, there may be an early critical period of development (common in TC and TT groups) with increased susceptibility to maternal EDC exposure. Specifically, the regions of impaired angio-follicular thyroid units (Gerard et al., 2000) with decreased NIS expression in thyrocytes lacking follicular organization suggest a lack of functional differentiation. We showed previously that coplanar PCB 126 and TCDD can down-regulate NIS transcripts in primary porcine thyroid cells (Pocar et al., 2006). However, this direct regulatory mechanism is unlikely to be responsible for focal lack of NIS expression as observed with our peri-conceptual exposure model. It seems conceivable to assume a developmental delay in thyrocyte differentiation upon exposure to ECs during an early critical time window.

Changes in distinct thyroid parameters were more pronounced in male fetuses. Male thyroids were exclusively affected in rats after maternal exposure to single high dose of TCDD at GD15 (Nishimura et al., 2003). Gestational exposure of rats to the strong estrogenic compound diethylstilbestrol (DES) (Yamamoto et al., 2003) increased TSH and T4 levels and resulted in thyroid hypertrophy in the male offspring. These data implicate the developing thyroid gland in the male, but not female, fetus to be more sensitive towards estrogenic environmental compounds. Indeed, we recently reported sex specific effects of ECs/EDCs in the human fetus (O’Shaughnessy et al., 2011). Fetal exposure to EDCs contained in sewage sludge was shown to disrupt fetal ovarian development (Fowler et al., 2008) and to cause significant changes in spermatogenesis in a subset of male fetuses (Bellingham et al., 2012) demonstrating that other endocrine systems were also affected in this sludge exposure model.

Despite the observed morphological alterations in fetal thyroid glands, plasma levels of free thyroid hormones fT3 and fT4 were unaltered in GD 110 ovine fetuses suggesting effective physiological compensation. A potential moderate hyperactivity of the pituitary-thyroid axis cannot be excluded as we were unable to measure fetal plasma TSH concentrations. The fetal HPT axis is functional during the last trimester of pregnancy (Obregon et al., 2007; Patel et al., 2011) and the increased weight of male fetal thyroid glands in the TC exposure group may reflect a thyroid glandular response to pituitary hyper-stimulation resulting in normal free hormone levels.

## 5 Conclusion

Peri-conceptual low-dose in utero exposure to a relevant complex mixture of environmental chemicals adversely affected cell proliferation, thyrocyte differentiation and the formation of intact angio-follicular units in the fetal thyroid. These changes may have long-term consequences for thyroid function during postnatal life.

## Figures and Tables

**Table 1:** Thyroid gland weights and plasma hormone concentrations of dams and fetuses.

Treatment groups	Constant exposure profile		Cross-over exposure profile	
	CC (n = 16F; *n* = 9 M)	TT (*n* = 13F; *n* = 13 M)	CT (*n* = 12F; *n* = 10 M)	TC (*n* = 13F; *n* = 11 M)
*Fetal lambs on day 110 (values are mean ± SEM)*				
Thyroids (mg); F	634 ± 31	647 ± 46	669 ± 41	602 ± 34
Thyroid weight normalized to body weight (F)	0.34 ± 0.01	0.36 ± 0.03	0.35 ± 0.01	0.33 ± 0.02
Thyroids (mg); M	565 ± 43	639 ± 35	592 ± 43	688 ± 55
Thyroid weight normalized to body weight (M)	0.29 ± 0.01⁎⁎	0.33 ± 0.02	0.33 ± -.01	0.36 ± 0.03⁎
fT3 (pmol/l); F	1.8 ± 0.1	1.9 ± 0.1	1.7 ± 0.1	1.8 ± 0.1
fT3 (pmol/l); M	1.8 ± 0.2	1.6 ± 0.1	1.6 ± 0.1	1.7 ± 0.1
fT4 (pmol/l); F	21.3 ± 0.6	22.2 ± 0.6	22.9 ± 0.7	21.2 ± 0.6
fT4 (pmol/l); M	20.5 ± 0.7	20.2 ± 0.6	20.9 ± 0.7	21.2 ± 0.7
ratio fT3/fT4; F	0.08 ± 0.005	0.08 ± 0.005	0.07 ± 0.004	0.08 ± 0.004
ratio fT3/fT4; M	0.09 ± 0.009	0.08 ± 0.003	0.08 ± 0.005	0.08 ± 0.003

*Maternal ewes on day 110 of pregnancy (values are mean ± SEM)*				
	CC (*n* = 10)	TT (*n* = 12)	CT (*n* = 10)	TC (*n* = 9)
Thyroids (g)	8.7 ± 0.7	9.0 ± 0.8	7.5 ± 0.5	7.9 ± 0.5
fT3 (nmol/l)	5.5 ± 0.4	5.6 ± 0.5	4.2 ± 0.3⁎	4.5 ± 0.3
fT4 (nmol/l)	17.3 ± 1.1	16.9 ± 0.6	13.5 ± 0.5⁎	13.9 ± 0.6⁎
ratio fT3/fT4	0.31 ± 0.01	0.33 ± 0.02	0.31 ± 0.01	0.32 ± 0.01
TSH (mIU/L)	0.14 ± 0.08	0.15 ± 0.07	0.02 ± 0.01	0.03 ± 0.01

⁎ *p* < 0.05 Compared to controls.

⁎⁎ *p* = 0.013 Between male and female controls for weights normalized to body weight.

**Table 2:** Blood vessel analysis comparing the blood vessel area distribution in four different size categories. Chi-Square analysis was performed to determine statistical significance (*p* < 0.05).

Changed in	*p*-Values	<100 μm^2^	100–200 μm^2^	200–400 μm^2^	>400 μm^2^
CT compared to CC	F: *p* = 0.13	↓	↑	↑	n.c.
	M: *p* = 0.31	↑	↓	↓	n.c.
	Comb: *p* = 0.66				

TC compared to CC	**^∗^F: *p*** **=** **0.007**	↓	↑	↑	↑
	**^∗^M: *p*** **=** **0.003**	↓	↑	↓	↓
	Comb*: p* *=* 0.0004				

TT compared to CC	**^∗^F: *p*** < **0.001**	↓	↑	↓	↑
	M: *p* 0.055	↑	↓	n.c.	n.c.
	Comb*: p* *=* 0.002				

TT compared to CT	**^∗^F: *p*** < **0.002**	↓	↑	↓	n.c.
	M: *p* 0.42	n.c.	↓	n.c.	n.c.
	Comb: *p* = 0.12				

TT compared to TC	**^∗^F: *p*** < **0.004**	↓	↑	↑	↓
	**^∗^M: *p*** < **0.001**	↑	↓	↓	-
	Comb: *p* = 0.38				

TC compared to CT	F: *p* 0.27	↓	n.c.	n.c.	↑
	**^∗^M: *p*** < **0.001**	↓	↑	↑	n.c.
	Comb: *p* = 0.05				

n.c. = No change.

**Table 3:** Percent distribution of the fetal thyroid blood vessel area in four size categories and in the four exposure groups CC, CT, TC, and TT.

Exposure group	Gender	<100 μm^2^	100–200 μm^2^	200–400 μm^2^	>400 μm^2^
CC	Female	89.73	6.80	2.46	1.01
	Male	92.41	5.67	1.56	0.35
	Combined	91.08	6.23	2.01	0.68

CT	Female	86.61	8.86	3.35	1.18
	Male	93.90	4.88	0.78	0.44
	Combined	90.04	6.99	2.14	0.83

TC	Female	85.65	8.57	3.47	2.31
	Male	88.85	8.48	2.58	0.08
	Combined	87.39	8.52	2.99	1.10

TT	Female	81.18	11.35	5.62	1.85
	Male	94.70	3.96	1.10	0.24
	Combined	88.49	7.35	3.17	0.98
